# Temporal and Spatial Cluster Analysis of 2019 Novel Coronavirus Pneumonia in Chongqing, 2020.1∼2020.2

**DOI:** 10.1155/2022/8491628

**Published:** 2022-09-16

**Authors:** Xiaoying Dai, Rui Sun

**Affiliations:** ^1^Chongqing Medical and Pharmaceutical College, Chongqing, China; ^2^Public Administration Bureau of Chongqing High Tech Zone Management Committee, Chongqing, China

## Abstract

In order to explore the spatial and temporal distribution characteristics of COVID-19 in Chongqing from January 22 to February 25, 2010, and provide a series of suggestions for scientific prevention and control of epidemic situation, we will mainly analyze the epidemic situation data of Chongqing Municipal Health Committee members and improve the descriptive analysis. Regional distribution and spatiotemporal scans were analyzed for COVID-19 outbreaks using ArcGIS10.2 and SaTScan9. 5 software. After the analysis, a total of 576 novel coronavirus pneumonia patients were confirmed in Chongqing. The incidence trend increased rapidly from January 22 to January 31, then decreased gradually, and there were no new cases until February 25. The purely spatial scanning results were consistent with spatiotemporal scanning, and a first-level accumulation area was detected by spatiotemporal scanning in the east and northeast of Chongqing from January 22 to February 10. From January 22 to February 25, 2020,COVID-19 occurred in the eastern and northeast regions of Chongqing. It is recommended to strengthen the detection of cluster areas to prevent another outbreak of COVID-19 risk.

## 1. Introduction

In recent years, biosecurity incidents have occurred frequently all over the world, which have brought serious threats to human life. Among them, SARS, which started in 2003, has brought great challenges to China's biological safety due to its rapid spread and high mortality, which has aroused widespread concern at home and abroad. An acute respiratory disease caused by a novel coronavirus first appeared in Wuhan in December 2019 [1]. After investigation, the gene sequence of this virus belongs to the same viral family as SARS and MERS. The disease mainly presents with fever, cough fatigue, respiratory failure, and other symptoms. Although the Chinese government immediately formulated the response to the epidemic prevention and control plan, such as blockade of Wuhan traffic, key crowd prevention and control, key places of prevention, and control measures, but due to the early outbreak during the Spring Festival travel, large-scale population flow had gathered to accelerate the spread of the virus, then they rapidly spread in China and even the world. On January 12th, 2020, the virus was officially named by the World Health Organization. On January 31, the WHO declared the novel coronavirus 2019 outbreak as a public health emergency of international concern, and characterized it as a global pandemic on March 11, 2020. As of May 29, 2022, China has reported a total of 2,139,725 confirmed novel coronavirus confirmed cases. On the same day, confirmed cases of COVID-19 rose to 525,299,796, causing 6,298,245 deaths. So far, tens of thousands of new cases remain in the United States, France, and Brazil. Today, the COVID-19 outbreak in China is being brought under control, but the global number of confirmed COVID-19 cases is still increasing rapidly, and the form of the epidemic continues to worsen. Therefore, all countries should work together to formulate effective measures to control the spread of the epidemic, and to understand the trend of the epidemic is also a key link in the epidemic prevention and control process [2].

Shortly after the outbreak, many scholars have done a lot of research on the novel coronavirus outbreak in 2019. These studies mainly focus on the genome characteristics, pathogenesis, clinical features, medical diagnosis and treatment of novel coronavirus [3, 4, 5]. In-depth research in this area is helpful to reduce the mortality rate and improve the cure rate of diagnosed patients. In addition, many countries will release the latest daily update of the epidemic through the official government platform, so that the public can quickly know the progress of the epidemic. However, these data do not give us a deep understanding of the temporal and spatial distribution of this epidemic. In fact, an in-depth analysis of the temporal and spatial correlation of novel coronavirus and its evolution law can play an important role in the analysis of the epidemic trend in the future and provide reliable prevention and control strategies for local governments.

As a mature statistical method, the spatiotemporal statistical method does not analyze the spatiotemporal distribution of confirmed cases, and explores the main gathering areas and visualization of diseases. Therefore, spatiotemporal statistical methods are widely used in the research of various infectious diseases [6, 7, 8, 9, 10]. Combined with the use of GIS software, it can intuitively reflect the changes of diseases and regional distribution. Angelababy [11], a domestic scholar, used a geographic weighted regression model and linear regression model, and found that the spatial distribution characteristics of 258 other cities showed spatial aggregation except Wuhan.

Baidu migration index (Wuhan migration) is statistically related to COVID-19, which indirectly shows that Wuhan has taken blockade measures, which greatly reduced the spread of the epidemic. Specifically, the study of COVID-19 found different spatial popular characteristics and different research emphases. For example, Xiong [12] used spatial analysis to study the spatial distribution of COVID-19 in Hubei Province, and found that there were differences in epidemic distribution between towns and cities in Hubei Province. The research on COVID-19 in Anhui Province is mainly concentrated in northern and central Anhui, and the spatial distribution of designated hospitals and epidemic areas is significantly different [13]. The number of confirmed cases in counties is mainly affected by population density, general public budget expenditure, and the distance from Wuhan. Bai [14] studied the temporal and spatial distribution characteristics of new coronavirus pneumonia in Zhejiang province and found that the time variation of new cases in Zhejiang province accords with the Pan-Poisson distribution function, and there is little difference between male and female susceptible infection rates. The infection rate of cases has obvious spatial aggregation, showing the overall pattern of “one belt and four hearts”. “One area” is the county-level regional unit of the coastal railway belt from Taiwan Province to Wenzhou, and the “four hearts” are Haishu District of Ningbo, Xiuzhou District of Huzhou, Shangcheng District of Hangzhou, and Jianggan District [15, 16]. It can be seen that different areas in COVID-19 have different epidemic trends and distribution characteristics. As COVID-19 is a new infectious disease, the spatial epidemic characteristics of such diseases are still being explored. It can be seen that different areas in COVID-19 have different epidemic trends and distribution characteristics. As COVID-19 is a new infectious disease, the spatial epidemic characteristics of such diseases are still being explored.

The spatial and temporal distribution of COVID-19 has been analyzed abroad, and the research using spatial analysis shows that the incidence of COVID-19 may be related to geographical location, altitude, and rainfall. Sub-Saharan Africa is currently at high risk of outbreak, and this situation may continue to worsen. From March 7th to May 22nd, 2020, all new coronavirus cases were collected in the northeast of Brazil, and this weekly incidence trend was found, which had a wide spatial autocorrelation in metropolitan areas [17, 18]. Further analysis shows that there are 11 spatial clusters, including 70 cities in Siirala, and the risk of epidemic spreading from cities to rural areas.

From the above analysis, we can find that different areas in COVID-19 have different epidemic trends and distribution characteristics. Therefore, understanding the temporal and spatial changes of COVID-19 in Chongqing and exploring the regional distribution of critical diseases are also important measures to effectively curb the epidemic spread in Chongqing.

## 2. Data and Methods

### 2.1. Research Area

Chongqing is located in the southwest of China, on the upper reaches of the Yangtze River, on the east edge of Sichuan Basin, bordering Hubei and Hunan in the east, Guizhou in the south, Sichuan in the west, and Shanxi in the north. The area is 470 kilometers long from east to west and 450 kilometers wide from north to south. The total area is 82,400 square kilometers. It is 39 times the total area of Beijing, Tianjin, and Shanghai. It is the largest city in China. By the end of 2014, Chongqing had 29 permanent residents. 914 million and 23 municipal districts, 11 counties and 4 autonomous counties. Chongqing has a large resident population, convenient transportation, and a large area, which makes the epidemic prevention and control more difficult. On January 21, 2020, the first case of novel coronavirus was diagnosed in Chongqing. 22 new cases were announced. On January 24th, according to the “Chongqing Special Emergency Plan for Public Health Emergencies”, Chongqing Municipal Government launched a Class I response to major public health emergencies and adopted the measures of home or residence isolation for 14 days and closed the management of key areas, which effectively cut off the spread of the epidemic and contained it. From January 21st to February 25th, the epidemic spread was stopped. Therefore, this paper collected the daily number of new cases in Chongqing from January 22 to February 25, 2020, once every seven days, using ArcGIS10. Analyze the spatial epidemic situation of COVID-19 in Chongqing, and use simple spatial scanning and spatial scanning to explore whether the epidemic situation in Chongqing is concentrated, so as to realize disease detection and early warning [19, 20].

### 2.2. Data Source

#### 2.2.1. New Confirmed Cases

The selected study samples were all obtained from the statistics of Chongqing Health Commission from January 22 to February 25, 2020 (daily new cases of COVID-19 in Chongqing). The data were manually collected, collated, and compiled into a dataset of confirmed COVID-19 cases in Chongqing.

#### 2.2.2. Demographic Data

Demographic data were collected from the permanent resident population of 38 county-level administrative regions in Chongqing in 2018.

### 2.3. Research Method

#### 2.3.1. Statistical Analysis

Excel 2003 and SPSS 19.0 software were used to summarize the number of new cases from January 22 to February 25, collate the data, and analyze the epidemic trend of the number of COVID-19 cases.

#### 2.3.2. Space-Time Aggregation Analysis

The spatiotemporal scanning statistics method is a window (space/time)-based scanning method. You can explore whether diseases are discrete or clustered in their temporal, spatial, or spatiotemporal distribution. Spatial scans analyze the data using dynamically changing cylindrical windows. Dynamic circular window radius represents the geographical location and regional size, in the study area of disease scanning area, under the Poisson distribution assumption, set 50% of the risk population as the maximum scan window, daily moving high time, the cylinder corresponding geographical location at the bottom, calculate the actual case and expected case calculation log likelihood ratio (LLR). The *P* value of the test statistics was calculated by Monte Carlo, and the number of Monte Carlo was generally set to 999 times. When the hypothesis test of LLR was *P* < 0.05, the difference in the relative risk value (RR) inside and outside the scan window can be considered statistically significant. The spatiotemporal cluster group with the maximum LLR value is defined as the main cluster area, and the rest are the secondary cluster area; the RR value indicates the estimated risk in the cluster area divided by the estimated risk in the cluster area. Spatiotemporal scanning, similar to the simple spatial scanning method, is also based on a cylindrical scanning window, but this window also considers time weights [21]. The cylindrical height indicates the time period of the clustering. In this paper, the confirmed cases from January 22 to February 25 will be grouped every seven days for a total of 5 groups. The weekly daily number of new cases of novel coronavirus was connected with the geographic information database of administrative districts and the resident population information database through the same field, and the geographic information database of five groups of cases was established to analyze the geographic distribution of weekly diseases. Simplicial spatial scan and spatiotemporal scan statistics were performed separately. The maximum spatial scan radius of 50% and the maximum radius of the temporal dimension of 50% were calculated by Monte Carlo using a discrete Poisson model to calculate LLR and RR for each scanned cylinder, exploring the clustering area classification results and visualized by Arc GIS10.

## 3. Results

### 3.1. Popular Overview

**Figure 1:**
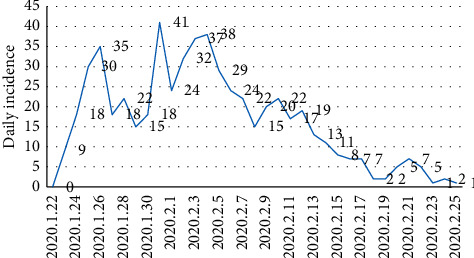
Daily number of COVID-19 patients in Chongqing.

From January 22nd to February 25th, the total number of confirmed cases of COVID-19 in Chongqing was 576. The number of cases of COVID-19 in Chongqing increased rapidly from January 22nd, reached the peak on January 31st, then decreased, with a small peak on February 4th, and finally decreased slowly to zero on February 25th. The details are shown in Figure 1.

### 3.2. Distribution Area

From January 22 to 25, the weekly incidence rate was 100,000, and the weekly incidence rate was 0 in other districts and counties in Chongqing except Beibei District. 1/100,000, the weekly incidence rate is 0.5/100,000,50 cases in the fifth week, 0.07/100,000 patients. Use the image rendering function of ArcGIS10.2. The weekly incidence rate (1/100,000) above 0 is divided into seven grades: 0, 0.0-0.4, 0.4-0.8, 0.8-1.2, 1.2-1.6, 1.6–2.0, and 2.0.

### 3.3. Spatial Scanning Analysis

Establishing case geographic information database in ArcGIS 10.2 Software is introduced into SatScan9.5 simple spatial scanning by the software shows that the incidence of COVID-19 in Chongqing is nonrandomly distributed, and the epidemic situation is mainly concentrated in the eastern and northeastern areas of Chongqing, as shown in Table 1 [26, 27, 28, 29, 30].

### 3.4. Space-Time Scanning

Based on the use of the ArcGIS10.2 software, the case geographic information database was established, and all the obtained results were imported into the SatScan9. 5 software to obtain the specific spatiotemporal scan results [31, 32, 33, 34]. From January 22 to February 10, the Chongqing coronavirus outbreak had obvious cluster characteristics, mainly concentrated in the east and northeast of Chongqing, and its location and simple space description of the cluster results are shown in Table 2.

**Figure 2:**
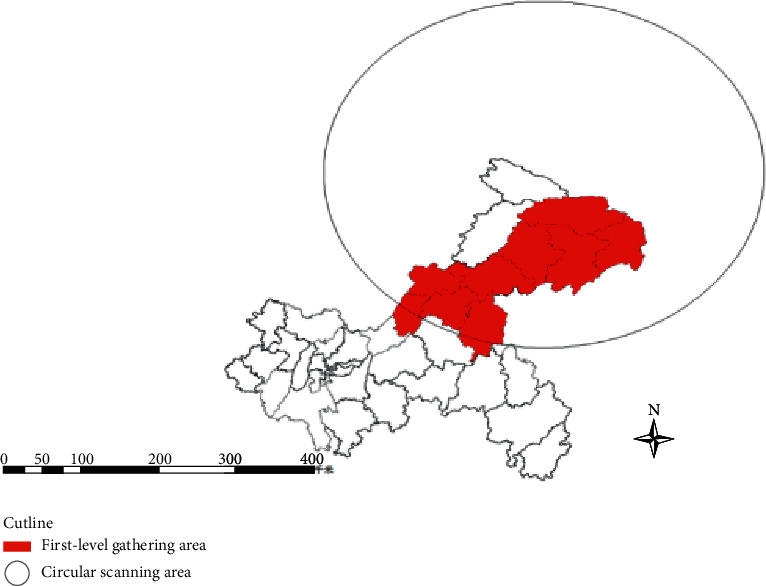
A schematic representation of the circular scan window from January 22 to February 25 was obtained based on the spatiotemporal scan statistical analysis.

We combine SatScan with GIS software for first-level cluster results of space-temporal scanning measurement analysis from January 22 to February 25 (Figure 2).

## 4. Conclusion

The COVID-19 pandemic has caused huge losses to the development of China and even the world. It not only harms the public property and health, but also has an indelible impact on the people's psychology [22, 23]. From the psychological perspective of some scholars have investigated the frontline medical personnel and college students and other groups in the form of questionnaires, advocating timely psychological counseling, and counseling for people of different professions [24, 25]. In the early days of the COVID-19 outbreak in 2020, a large number of migrant workers returned to Chongqing from Hubei province, which is bound to make Chongqing the worst-hit area of imported cases from Hubei province. It can be seen from the epidemic situation and regional distribution that in the first and second weeks after the first confirmed case appeared in Chongqing on January 21, the number of epidemic cases has increased rapidly, accounting for 63.5% of the total number of cases. The number of COVID-19 decreased slowly from February 4, and no new cases for 14 consecutive days by February 25, indicating that the prevention and control measures for COVID-19 in Chongqing are effective and reliable [26].

From the perspective of spatiotemporal analysis, based on the data of COVID-19 confirmed cases released in Chongqing, the COVID-19 epidemic geographic database was established. The geographic database data are imported into SatScan9.5 software for spatiotemporal scanning analysis to detect the distribution of spatial and temporal aggregation, and determine whether the disease aggregation is statistically significant. Apart from the traditional epidemiology, it can only describe its characteristics from the three diseases, while ignoring the law of spatial and temporal distribution [27]. Due to the limited data collection information, simple spatial scanning and spatiotemporal scanning were mainly adopted. As can be seen from the simple spatial scanning results, the incidence of COVID-19 in Chongqing showed a nonrandom distribution, with a major cluster area existing every week. From January 22 to 28, the first gathering area mainly included districts and counties: Liangping District, Wuxi County, Yunyang County, Fengjie County, Wanzhou District, Kaizhou District, Zhongxian County, Wushan County, Dianjiang County, and Shizhu Tujia Autonomous County [28]. From January 29 to February 4, the first gathering area was Wanzhou District, and from February 5 to 11 was the same as last week, still Wanzhou District. From February 12 to 18, the first gathering area included Wanzhou District, Zhongxian County, Yunyang County, and Fengjie County. From February 19 to 25, the first gathering area included Wanzhou District, Kaizhou District, Zhongxian County, Fengjie County, and Wuxi County. As can be seen from the above that from January 22 to February 25, the main cases were concentrated in the east and northeast of Chongqing [29, 30, 31]. It is speculated that these areas border Hubei, resulting in more imported cases compared with other regions, and the epidemic spread rapidly, among which Wanzhou District, bordering Hubei, is the most significant [32, 33, 34, 35]. Spatiotemporal scanning adds a time dimension on the basis of pure spatial scanning, which can make up for the lack of time dimension to a certain extent. The results of the space scanning is similar to simple space scanning results, on January 22, 2020 to February 10 during this period is a primary area, including Wuxi, Liangping District, Yunyang, Fengjie District, Wanzhou District, Zhongxian, Wushan, Dianjiang, and Tujia Autonomous County, prompt after the outbreak should also strengthen the disease monitoring of the region, prevention and control of the outbreak in the area of outbreak again [36, 37].

Conditions of limitations mainly are as follows: first, the data used in this study are mainly derived from the case data published by Chongqing Health Commission, and the content is not detailed enough. Therefore, this paper can only discuss the spatial or temporal distribution of the epidemic and cannot further judge which factors can affect the development of the epidemic. Second, at present, Chongqing is only taken as the research area, and the spatial association with other provinces and cities is not considered, or the differences or association between regions within Chongqing is not considered. Third, the spatiotemporal scanning method assumes that the window is round, while in practice, the shape of the scanned region may be irregular, with some error with the assumption, which may cause some bias in the results.

## Figures and Tables

**Table 1:** First-level clustering results based on the analysis of simple spatial scan statistics.

Time quantum	Gather from the number	Clustering area name	Actual number of onsets	Expected number of morbidities	RR	*P*
January 22nd–January 28th	10	Liangping District, Wuxi County, Yunyang County, Fengjie County, Wanzhou District, Kaizhou District, Zhongxian County, Wushan County, Dianjiang County, and Shizhu tujia Autonomous County	77	37.02	24.76	= 0.001

January 29th–February 4th	1	Wanzhou District	48	11.64	35.04	= 0.001

February 5th–February 11th	1	Wanzhou District	36	7.39	31.77	= 0.001

February 12th–February 18th	4	Wanzhou District, Zhongxian County, Yunyang County, Fengjie County	17	6.52	7.19	0.019

February 19th–February 25th	5	Wanzhou District, Kaizhou District, Zhong County, Fengjie County, and Wuxi County	12	3.00	7.99	= 0.001

**Table 2:** First-level and clustering results based on spatiotemporal scan statistics analysis.

Time quantum	Gather from the number	Clustering area name	Actual number of onsets	Expected number of morbidities	RR	*P*
January 22nd ∼ February 10th	9	Wuxi County, Liangping District, Yunyang County, Fengjie County, Wanzhou District, Zhongxian County, Wushan County, Dianjiang County, and Shizhu Tujia Autonomous County	212	72.34	109.7	= 0.001

## Data Availability

The dataset used during the current study are available from the corresponding author on reasonable request.
